# Two matched filters and the evolution of mating signals in four species of cricket

**DOI:** 10.1186/1742-9994-6-22

**Published:** 2009-09-28

**Authors:** Konstantinos Kostarakos, Matthias R Hennig, Heiner Römer

**Affiliations:** 1 Zoology, Karl-Franzens-University, 8010 Graz, Austria; 2 Department of Biology, Humboldt-Universität zu Berlin, 10115 Berlin, Germany

## Abstract

**Background:**

Male field crickets produce pure-tone calling songs to attract females. Receivers are expected to have evolved a "matched filter" in the form of a tuned sensitivity for this frequency. In addition, the peripheral directionality of field crickets is sharply tuned as a result of a pressure difference receiver. We studied both forms of tuning in the same individuals of four species of cricket, where *Gryllus bimaculatus *and *G. campestris *are largely allopatric, whereas *Teleogryllus oceanicus *and *T. commodus *occur also sympatrically.

**Results:**

The sharpness of the sensitivity filter is highest for *T. commodus*, which also exhibits low interindividual variability. Individual receivers may also vary strongly in the best frequency for directional hearing. In *G. campestris*, such best frequencies occur even at frequencies outside the range of carrier frequencies of males. Contrary to the predictions from the "matched filter hypothesis", in three of the four species the frequency optima of the two involved filters are not matched to each other, and the mismatch can amount to 1.2 kHz. The mean carrier frequency of the male population is between the frequency optima of both filters in three species. Only in *T. commodus *we found a match between both filters and the male carrier frequency.

**Conclusion:**

Our results show that a mismatch between the sensitivity and directionality tuning is not uncommon in crickets, and an observed match (*T. commodus*) appears to be the exception rather than the rule. The data suggests that independent variation of both filters is possible. During evolution each sensory task may have been driven by independent constraints, and may have evolved towards its own respective optimum.

## Background

Sensory pathways serve to extract information from the physical environment. On this basis an organism may choose an appropriate behavioural action that ultimately maximizes its fitness. Sensory systems and their underlying neuronal processing capacity are therefore - in a general sense - tuned to the relevant physical environment [1] and may be aided by rather peripheral 'matched filters' that relax the nervous system from computational strain [2]. If matched filters and sensory pathways are involved in signal processing in communication systems, a close and specific match between the physical properties of the sender's signal and the receiver's filter are expected, because it enables reproductive isolation by facilitating interspecific mate recognition [3,4]. A comparison across different species that employ signals of the same modality for communication are consequently likely to reveal interspecific differences, but nevertheless a maintained match between sender and receiver.

More specifically, the "matched filter hypothesis" has been put foreward for acoustic communication systems of anurans [5,6] by arguing that receivers should gain an advantage from being selectively tuned to the vocalisation frequencies, since the match between the sensitivity of their auditory system and the energy spectrum of the sender's vocalisation would maximize the signal-to-noise-ratio for reception. Experimental evidence for this hypothesis was obtained for frogs [7,8]. Similarly, the acoustic communication of crickets provides another outstandingly clear case in point, since the male calling song is a rather pure tone (with higher harmonics at lower intensity) produced by modified forewings [9]. The auditory periphery is - in the frequency range used exclusively for conspecific communication - narrowly tuned to a single frequency, which is expected to match the highly resonant frequency spectrum of the male song [10].

A second matched filter exists in the cricket's auditory system which is related to the task of sound localisation. Since crickets (or other small animals) cannot only rely on diffraction of sound around the head or body when using lower frequencies with larger wavelength, the necessary interaural intensity differences (IIDs) result from a pressure difference receiver with a functional three-input system for the sound, provided by a system of connecting trachea between the ears in the forelegs, and a phase delay mechanism [11-14]. This mechanism is inherently sharply tuned, so that reasonable IIDs in the order of 5dB in front of the cricket for sound localization are only provided for a narrow range of frequencies [15]. Thus, there are two known sharply tuned frequency-selective filters in the receiver, which provide a sensory representation with a high signal to noise ratio, and from which information about the 'what' and 'where' can be extracted [16].

However, contrary to the prediction of matched filters inherent to this term a recent study of signals and receivers in the cricket species *G. bimaculatus *revealed a considerable mismatch not only between the frequency of the signal and the tuning of the receiver's auditory system, but also between the two filters that serve the tasks of 'what' and 'where' (i.e. detection and localization [17]).

Conceivable causes for the observed variation in the tuning of the receiver and its consequences for the frequency content of the senders' signal in crickets lie in the evolutionary history of acoustic communication within a rather narrow frequency band and the trade-offs that arise from ecological settings. Since further insights into the causes and consequences of the observed mismatch can be obtained by a comparative approach, we here investigated the frequency spectra of the signal and the filter properties of both involved filters in four species of crickets that differ in their frequency tuning, by using the spiking activity of the ascending neuron AN1 as a read-out for frequency tuning. Generally we surveyed whether such a mismatch is a peculiarity of *G. bimaculatus *or a general feature of the auditory periphery in crickets. Specifically we examined (1) whether signals are always driven to the frequency mean between the receivers' sensory filters, (2) whether there is a consistent pattern to the mismatch visible by a comparison of different species and (3) whether interindividual variability in tuning and frequency between the filters for detection and localization can provide clues about the constraints that give rise to the observed mismatch during evolution.

## Results

### Sensitivity

**Figure 1:**
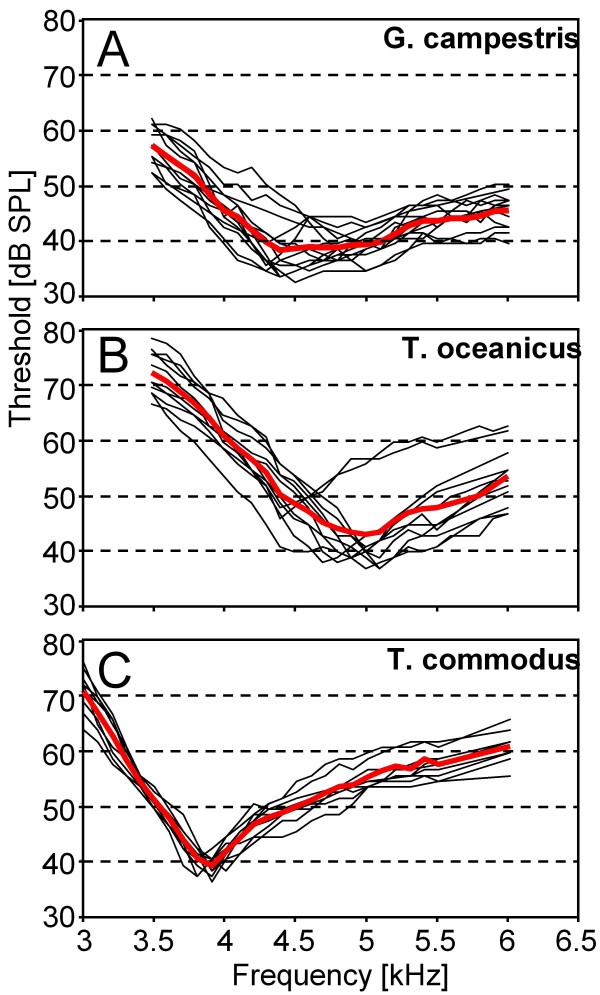
**Sensitivity tuning of the AN1-neuron in three species of field crickets (*G.c*.: N = 14; *T.o*.: N = 11; *T.c*.: N = 9)**. The mean tuning curve, representing the population mean sensitivity of receivers, is shown in red.

The tuning of the sensitivity of the AN1-neuron represents a filter, which strongly predicts the amount of afferent excitation in female receivers for male calling songs varying in CF. In a previous study on G.b. we have described the amount of variation in sensitivity and tuning [17], and comparative data are shown in Fig. 1A-C for all individuals in *G.c., T.o*. and *T.c*. AN1 in *G.c*. is tuned to best frequencies between 4.3 to 5.1 kHz, with lowest thresholds between 32 to 40 dB SPL. Nine of eleven individuals in *T.o*. exhibited a tuning of AN1 between 4.5 to 5.1 kHz, but two individuals were tuned at 4.4 kHz, and were significantly less sensitive at higher frequencies. *T.c*. is the species with the least variation in AN1-tuning, with all specimen tuned between 3.8 to 4 kHz and rather similar shape of the tuning curve, and thresholds between 36 and 40 dB SPL.

**Figure 2:**
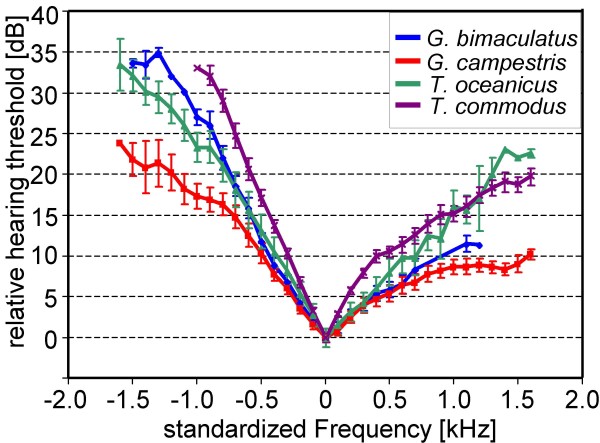
**Standardized average tuning of the AN1-neuron in four species of field crickets**. The best frequency in each individual and species was set at 0 Hz, and the higher thresholds to lower and higher frequencies averaged accordingly.

Figure 1A-C also includes the average AN1-tuning curve for each species, representing the mean sensitivity values of receivers within the population for the investigated frequencies. However, due to the variation of the best frequency in individual receivers, this average tuning curve does not reflect the average shape of the AN1-tuning in individuals. Figure 2 therefore represents the standardized tuning curves of the four species, with the best frequency in each individual and species set at 0 Hz, and the higher thresholds to lower and higher frequencies averaged accordingly. In this way, the standardized tuning curves represent the mean shape of AN1-tuning in the four species. In general, all species show a higher roll-off towards lower compared to higher frequencies. This increase in threshold towards lower frequencies is 33 dB for *T.c*., 27 dB for *G.b*., 23 dB for *T.o*., and 17 dB for *G.c*. with 1 kHz deviation from the best frequency. Similarly, towards higher frequencies the roll-off is 15 dB for *T.c*., 11 dB for *G.b*., 16 dB for *T.o*., and 9 dB for *G.c*. with 1 kHz deviation from the best frequency. Clearly, *T.c*. exhibits the most selective, and *G.c*. the least selective tuning. Quantitative values concerning variation in sensitivity and tuning are summarized in Table 1.

**Table 1:** Parameters characterizing the frequency tuning of the AN1-neuron in the four species of field cricket.

	**Best Frequency of mean tuning curve [kHz]**	**Mean threshold [dB SPL]**	**Best frequency of individual tuning curves ± SD [kHz] [Range of variation]**	**Mean width of tuning 5dB above best frequency ± SD [Hz] [Range of variation]**	**Mean CF of male signal ± SD [kHz] [range of variation]**
Gryllus bimaculatus	4.9	41.8	4.5 (± 0.23) [4-5.1]	660 (± 291) [300-1100]	4.7 (± 0.21) [4.3-5.2]

Gryllus campestris	4.4	35.1	4.6 (± 0.27) [4.3-5.1]	757 (± 262) [300-1400]	4.7 (± 0.2) [4.3-5.1]

Teleogryllus oceanicus	5.0	39.9	4.8 (± 0.26) [4.4-5.1]	518 (± 194) [200-900]	4.8 (± 0.21) [4.3-5.4]

Teleogryllus commodus	3.9	37.9	3.9 (± 0.3) [3.4-4.5]	267 (± 100) [100-400]	4.0 (± 0.15) [3.8-4.2]

### Directionality

**Figure 3:**
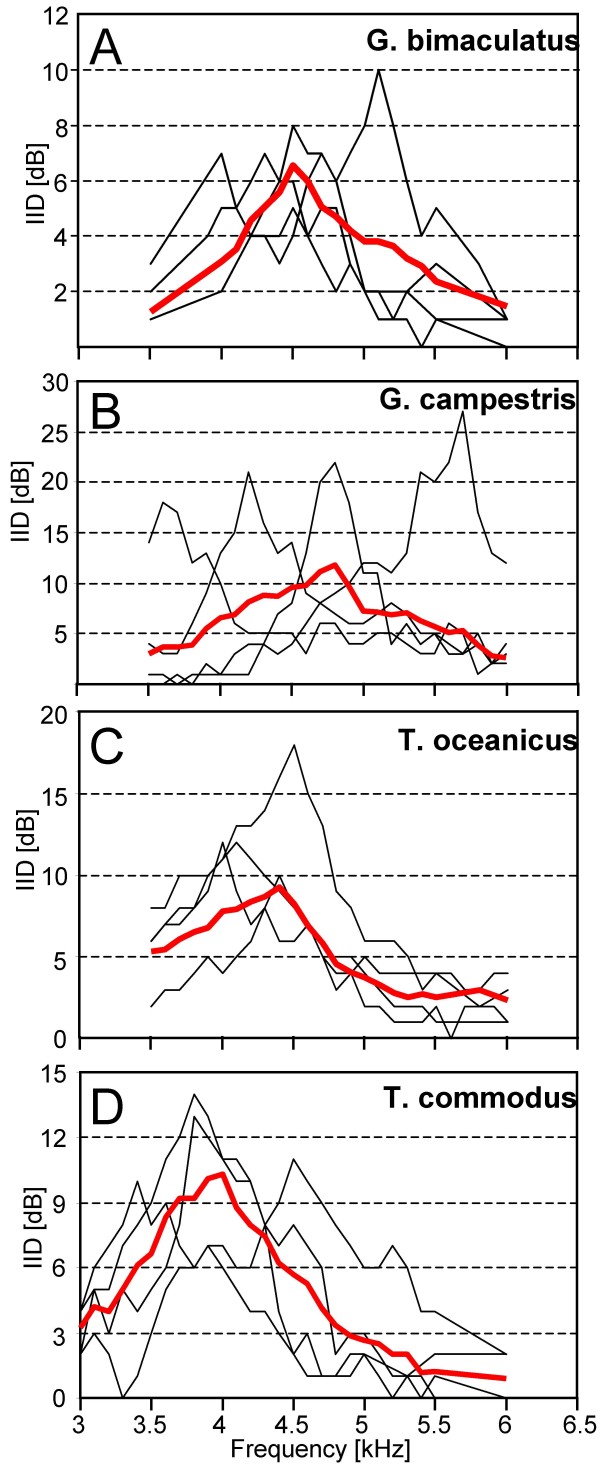
**Directional tuning in four individuals of each species of field crickets**. Note the interindividual variation in the optimum frequency providing largest IIDs, in particular in *G.c*. and *G.b*., and the difference in the absolute amount of IIDs between species. The population mean directionality of receivers is shown in red. For further explanation see text.

Directional hearing in field crickets is based on a pressure difference receiver with a functional three-input system for the sound, provided by a complicated anatomical arrangement of connecting trachea between the ears in the forelegs, and a phase delay mechanism [11,12,14]. For *G.b*. it was shown, that this directionality is sharply tuned, so that IIDs high enough for the localization of a calling male are only provided for a narrow range of frequencies [14,17]. Here we examined the peripheral directionality in all four species, and in the same individuals from which the frequency tuning curves were examined. Figure 3A-D shows representative examples of such directional tuning in four individuals in each species, to demonstrate the amount of inter-individual variability with respect to the best frequency, and the absolute values of IIDs provided by the directionality. In *G.b*., the IID optimum varies from 4.0 to 5.1 kHz in different individuals, with maximum values ranging between 5 to 13 dB. The largest variation in the optimum IID was found in *G.c*. (Fig. 3B), ranging from 3.6 to 5.7 kHz. In addition, this species also exhibited the largest IIDs of up to 27dB at the best frequency. The variation of best frequencies is smaller in *T.o*. and *T.c*. (Fig. 3C, D). Quantitative values concerning variation in directional tuning of the four species are summarized in Table 2.

**Table 2:** Parameters characterizing the peripheral directionality in the four species of field cricket.

	**Optimum of mean directional tuning [kHz]**	**Max. IIDs of mean standardized tuning curve [dB]**	**Optimum of directional tuning in individuals [kHz]** **[Range of variation]**	**Mean width of directional tuning 5dB below optimum ± SD [Hz]** **[Range of variation]**	**Mean CF of male signals ± SD [kHz]** **[Range of variation]**
Gryllus bimaculatus	4.5	7.7	4.5 (± 0.23) [4-5.1]	1395 (± 488) [400-2200]	4.7 (± 0.21) [4.3-5.2]

Gryllus campestris	4.8	17.4	4.6 (± 0.56) [3.6-5.7]	567 (± 602) [100-1900]	4.7 (± 0.2) [4.3-5.1]

Teleogryllus oceanicus	4.4	11.2	4.2 (± 0.27) [3.6-4.5]	991 (± 591) [200-2500]	4.8 (± 0.21) [4.3-5.4]

Teleogryllus commodus	4.0	13	3.9 (± 0.3) [3.4-4.5]	833 (± 559) [200-1800]	4.0 (± 0.15) [3.8-4.2]

**Figure 4:**
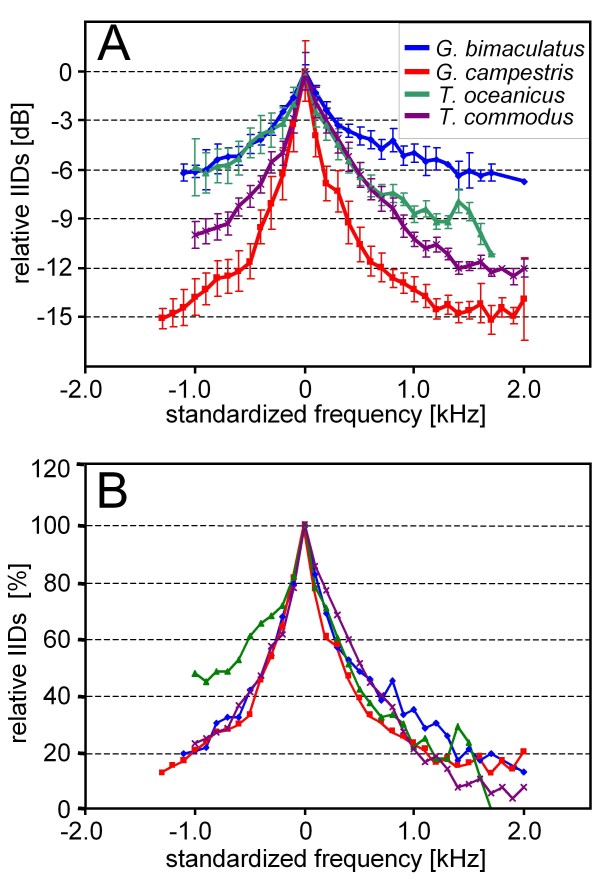
**Standardized average directional tuning in four species of field crickets**. A) The optimum frequency in each individual and species was set at 0 Hz, and the IIDs to lower and higher frequencies averaged accordingly. B) The same data as in A) normalized in percent of the maximum IID.

Figure 3A-D also shows the average directional tuning for each species, which represents the mean IIDs of receivers within the population for the investigated frequencies. However, as with the average frequency tuning curve of AN1, due to the high variation of the best frequency in individual receivers (in particular for *G.c*.), this average directional tuning underestimates the amount of IIDs available for individual receivers for directional hearing. We therefore standardized the directional tuning in Fig. 4A, by setting the frequency of maximal IID in each individual and species at 0 Hz, and averaging the decreasing IIDs for each frequency step accordingly. In Fig. 4B the data on directional tuning are further standardized by setting the maximum IID achieved in each species to 100 percent.

The decrease of IIDs above and below the directional optimum is almost symmetrical for *G.b., G.c*. and *T.c*., whereas for *T.o*. the roll-off towards higher frequencies is steeper. However, there are large differences with respect to the amount of IIDs available, and thus also the steepness of the directional tuning. For a shift of only 500 Hz towards lower frequencies away from the optimum frequency, IIDs are reduced by 11.6 dB in *G.c*., 7.6 dB in *T.c*., 4.5 dB in *G.b*. and 4.4 dB in *T.o*. For further quantitative differences in directional tuning between the four species see table 2.

### Two matched filters in each of the four cricket species

**Figure 5:**
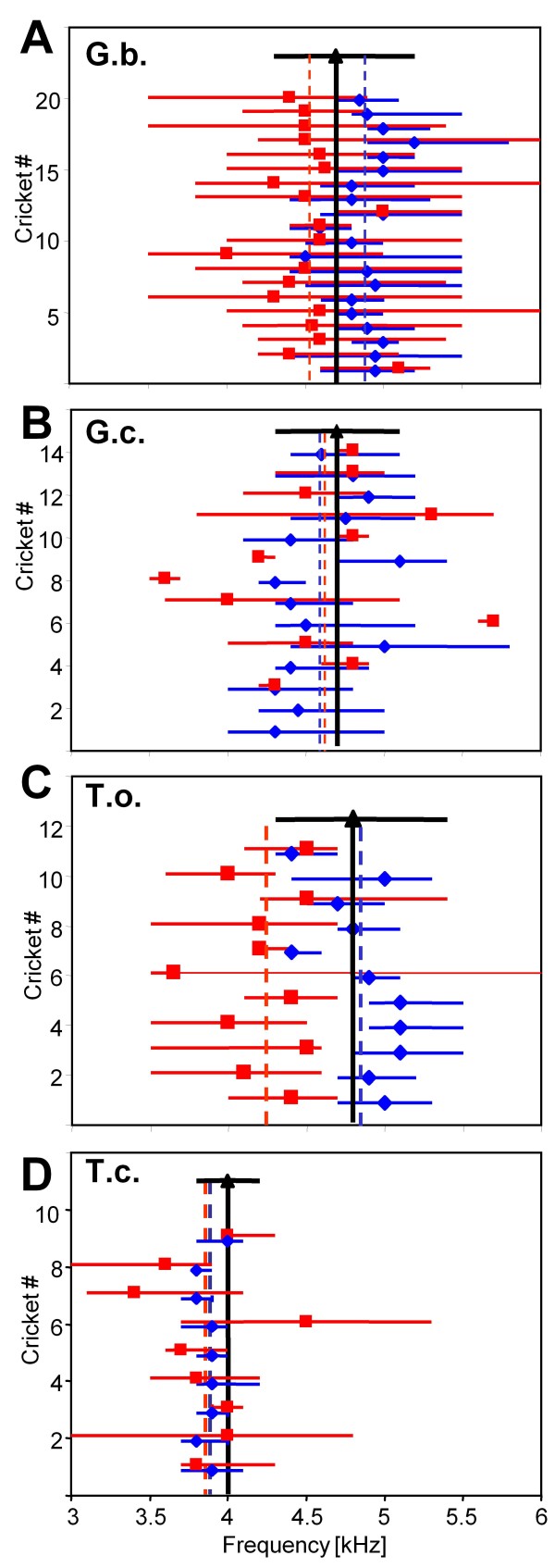
**Interindividual variation of sensitivity tuning and directional tuning in four species of field crickets**. For each individual, the best frequency in tuning and the width of the tuning curve 5 dB above the best frequency (blue diamonds and horizontal bar) as well as the optimum of directional tuning and the width of the tuning curve 5 dB below the optimum (red square and horizontal bar) are shown. In addition, the mean values for sensitivity and directionality are given as dashed, vertical blue and red lines, respectively. The range of variation, and the mean value, of the male CF is shown as horizontal and vertical black bars, respectively.

The neuronal tuning of AN1 and the directional tuning of the peripheral auditory system represent matched filters which, ideally, should peak at the same best frequency. However, an inspection of tuning curves in Fig. 1 and 3 already indicates that such a match does not necessarily exist. Since we investigated both tunings in the same individuals, we could assess this match for each receiver, and describe its intraspecific variability. These data are summarized for all four species of field cricket in Fig. 5A-D, where for each individual the best frequency of the AN1-tuning (blue diamonds), and the best frequency of the peripheral directionality (red squares) are shown, together with the measure of sharpness of tuning for both (blue and red horizontal bars, respectively). For *G.b*. (Fig. 5A) a match between both filters does exist in only 2 out of twenty individuals. In seventeen individuals the optimum in directionality is shifted considerably towards lower frequencies; in only one individual the optimum in directionality was 150 Hz above the best frequency of the AN1-tuning. As a result of this mismatch in most individuals, the two filters differ by 400 Hz (4.5 kHz compared to 4.9 kHz) with respect to their best frequency (Whitney rank sum test, P < 0.001). Included in Fig. 5 is also the range of the distribution of CFs of the male population, and its mean value (black horizontal bar and triangle). The mean CF in *G.b*. is at 4.7 kHz, and thus exactly between the best frequencies of the two involved matched filters.

The situation in *G.c*. (Fig. 5B) appears to represent a perfect case for a match between the two "matched filters", since the mean best frequency of individuals in tuning and in directional hearing is almost identical at 4.6 kHz, and the mean CF in the population closely matches this value. However, at the individual level, only two of twelve show this match, whereas in others, large differences exist between these filters, which can amount to 1.2 kHz. Furthermore, there is no consistent trend - such as in *G.b*. - for the best frequency in one filter to be above or below the other; rather, directionality can peak 1.2 kHz above the sensitivity of AN1 (cricket # 6) but 900 Hz below in another individual (cricket # 9). Due to the high variability of the individual filters there is no significant difference between the two frequency optima, (T-test, P = 0.95). Moreover, the best directionality in some individuals is far outside the range of variation of CF within the population, and the best sensitivity of AN1-tuning often coincides with the borders, rather than the mean, of the calling song distribution.

*T. oceanicus *exhibits the greatest disparity between the optima of the two matched filters of all four cricket species examined (Fig. 5C), and the difference is significant; (T-Test, P < 0.001). The average AN1-tuning for all 11 individuals is best at 4.85 kHz, whereas the mean directionality peaks at 4.2 kHz; in two of eleven individuals there was a disparity of 1.1 kHz (cricket # 4) and 1.2 kHz (cricket # 6). Notably, there is an almost perfect match between AN1-sensitivity and the mean CF of the male calling song, but the best directionality is at a frequency of 4.2 kHz, and thus just outside the range of CFs produced by males.

*T. commodus *is the species with a perfect match between both matched filters, which is evident in the means of the frequency optima, but also in the individuals (Fig. 5D; therefore no significant difference between both optima; Whitney rank sum test, P = 0.825). In seven of nine crickets the disparity between both filters was only 200 Hz or less. Furthermore, the best frequency in both filters comes close to the mean CF of male calling songs at 4 kHz. Finally, as already obvious from the individual tuning curves in Fig. 1C, and the standardized tuning curve in Fig. 2, *T.c*. exhibits the most selective tuning curve in all individuals examined (Table 1).

**Figure 6:**
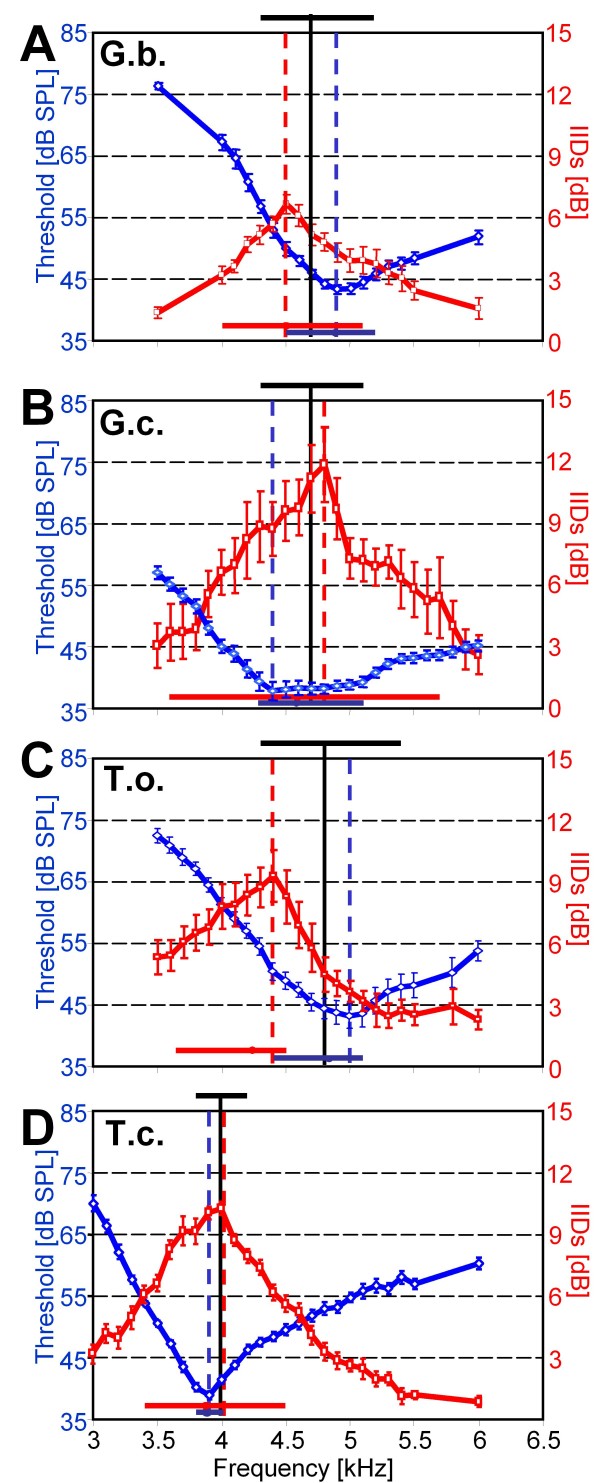
**Summary of sensitivity tuning and directional tuning in four species of field crickets**. A) modified from [17]. For each species, the mean sensitivity tuning (blue) and directional tuning (red) is shown for comparison. The respective best frequencies for these traits from these averaged curves are shown as blue and red dashed vertical lines. The range of variation in the respective best frequencies for individuals is given as horizontal blue and red bars, respectively. Similarly, the range of variation, and the mean value of the male CF are shown as horizontal and vertical black bars, respectively.

Figure 6 summarizes the above findings, by combining the averaged tuning curves (blue) and the directional tuning (red) with the range of calling song CFs which females can experience (black bar on top), and also with the degree of variation present in the best frequency and best directionality in individuals (blue and red bars below, respectively). In three species (*G.b., G.c*. and *T.o*.) the mismatch between both filters is obvious, and is largest in *T.o*. In these three species, the mismatch in G.c. is different from the other two species since the directional optimum is shifted towards higher frequencies, whereas in *G.b*. and *T.o*. it is shifted towards lower frequencies relative to the best frequency in tuning. The range of variance of the directionality optimum is significantly larger compared to the range of the frequency tuning in AN1 in *G.c*. and *T.c*.

## Discussion

Our results demonstrate that a mismatch between the sensitivity and directionality tuning is not uncommon in crickets (Figs. 5, 6), and an observed match (*T.c*.) appears to be the exception rather than the rule. For all four species the mean and distribution of the frequency of the male signal was found at intermediate values (Figs. 5, 6). From our data set, two main questions arise: (1) what are the consequences for the males, i.e. at which frequency should they signal given the divergent tuning of the sensory structures of the receiver? (2) Why is there a difference in the receiver tuning with respect to sensitivity and directionality?

### Male strategy and signal design

Males apparently signal at an intermediate frequency and may therefore try to activate both the sensitivity filter and the directional filter with similar magnitude (Figs. 5, 6). A tuned sensitivity filter should result in strong stabilizing selection, i.e. all males should principally signal at the sensitivity maximum of females in order to enhance the range of their signal and thus attract more females. However, as our data suggest there is a trade-off due to the mismatch of both filters in three of the four cricket species, since there is a loss in the ability of females to localize sound at the frequencies which activate the frequency filter strongest. For example, if males signal at the best frequency of AN1-tuning, females experience a significant reduction of IIDs necessary for localisation (*G.b*. = 3.7 dB; *G.c*. = 9.5 dB; *T.o*. = 7 dB; *T.c*. = 2.9 dB). Vice versa, if males signal at the optimal IID-frequency, there is a considerable loss in terms of relative signal loudness (and thus range): *G.b*. = 8.9 dB; *G.c*. = 4.7 dB; *T.o*. = 15.6 dB; *T.c*. = 2.9 dB. In order to judge the relevance of these trade-offs we have to consider, however, the absolute values available in the different species: A reduction of 1dB IID is much more relevant for *G.b*. than for *G.c*., because the average optimal amount of IID is only 7.7 dB in *G.b*., but 17.4 in *G.c*. In addition, the strong interindividual variation between females with respect to the best frequencies in sensitivity and directionality could also soften the selection pressure on males for calling at particular frequencies, since for almost each male CF there will be female receivers being activated with reasonable sensitivity and directionality.

### Is the trade-off between sensitivity and directionality detrimental for phonotaxis?

The directional tuning shown in Figs. 3 and 6 would suggest that IIDs in the order of 3 dB or more are available for an orienting female even when listening to a calling song with a CF differing from the best directionality. Given that in crickets, bushcrickets and grasshoppers IIDs of 2-3 dB are sufficient to evoke reliable turns towards the side where the sound level is highest [18-20], and these IIDs are reliably encoded in pairs of directionally sensitive interneurons [21] one may argue that the observed mismatch might not be detrimental at all. However, these upper limits of resolution of IIDs were obtained in approaches using precisely controlled acoustic conditions or in dichotic stimulus paradigms. How well the proximate mechanisms of directional hearing do work in real habitats is only poorly known. A strong degradation of directional hearing by bushcrickets and grasshoppers in dense vegetation was observed indicating that multiple reflections and other deviations from a free sound field produce severe degradation of directional cues [22,23]. Michelsen and Rohrseitz [24] observed that the scatter of the phase part of directional cues was not as dramatic close to the ground as that of the amplitude part. They concluded that directional hearing in crickets on the ground is not severely impaired, because it is dominated by the phase relationship of the three sound inputs of the pressure difference receiver [24]. However, a recent neurophysiological study in a natural habitat of *G.c*. demonstrated a severe degradation of directionality, as measured in the amount of bilateral discharge differences in the pair of AN1-neurons (Kostarakos and Römer unpublished). This is remarkable since *G.c*. is the species with the largest IIDs of all 4 species tested (Fig. 4), and it would indicate that in the natural situation in which communication takes place, the mismatch of both filters indeed matters and can be detrimental for an orienting female.

In *G.c*., males signal close at the IID-peak, a property that may be understood by inspection of the sensitivity tuning; since the tuning of the population mean is rather broad due to the high interindividual variability over a wide frequency range (Fig. 1), males may rather gain by signalling with a frequency close to the directionality optimum for which high IIDs are available (Figs. 4, 5). It should be noted, however, that these values may be quite different, if we consider the variation of the two filters in individuals. In particular, females of *G.c*. exhibit rather different frequency optima in directional hearing, ranging from 3.6 to 5.7 kHz (Fig. 3B). No male was found singing at these extreme frequencies (Fig. 5B, 6B). If males would signal at the population mean frequency of the calling song at 4.7 kHz, the females with these extreme frequency optima in directional hearing would experience a loss in directionality of 19 dB and 12 dB, respectively.

### The mismatch between sensitivity and directionality

Even if the mean values of these two optima overlap almost perfectly, and are consistent with the mean CF of the male calling song (as in *G.c*.; Fig. 5B), this is not the case at the individual level, where large deviations between both optima occur. This points to the importance of studying the variation in such receiver traits at the individual level, which has been largely neglected in the past (but see e.g. [25]). Furthermore, there is apparently no systematic link between the two optima which would point to a common underlying mechanistic cause, e.g. the sensitivity optimum is not always higher than the directionality optimum, since in one species both optima are reversed (*G.c*.). Similarly, the interindividual variation within each species revealed deviations in both directions (Fig. 5) and therefore makes a correlation with a simple morphometric measure such as body size or length of tracheal tube unlikely. Consequently, the data suggests that independent variation of both optima is possible. During evolution each sensory task may have been driven by independent constraints, and may have evolved towards its own respective optimum. However, then one is faced with the paradoxical question: if both, sensitivity and directionality may evolve independently, why do receivers fail to make both optima congruent such that a match with the males' signal is obtained and their own resources/constraints (energy and risk of phonotaxis towards the male signal) are optimized?

Given the observed variation between females within each species, the observed distribution of song frequencies of males strongly favours the idea that the population tuning of both peripheral filters drive the CF of male songs to intermediate/mean values. From a male point of view the number of responding females and thus potential mates would be maximised, although a certain quality of both available cues is sacrificed. Interestingly, the observed male strategy suggests a different point of view on the concept of 'matched filters'. By principle, 'matched filters' are thought to *match *properties of the physical environment in order to relieve the nervous system from processing tasks [2]. However, our data would suggest that matched filters in the auditory system of crickets appear to drive the frequency distribution of the signal to match the filter rather than vice versa. Although this reversal may be related to the dedication of acoustic communication in crickets that exclusively evolved for processing of conspecific acoustic signals, it rather reflects the selection by female choice that acts on communication signals [26,27].

### Evolutionary trends and constraints

From an evolutionary point of view there appear to exist two hypotheses that may explain the recent paradox in sensory tuning in crickets: (1) the independent variation of both traits in crickets may be explained by the original evolutionary constraints under which acoustic communication in crickets evolved, (2) local adaptation of allopatric and sympatric species - due to relatively recent speciation events - may explain the presently observed frequency values of the two traits in question.

Acoustic communication in crickets probably evolved for interactions during mating from which the original constraints on auditory structures arose [27,28]. For predator detection, originally cercal structures and later, sensitivity for ultrasonic signals emitted from bats were employed [29,30]. Thus, the sense of hearing in crickets served intraspecific communication exclusively and may have been tuned to a given frequency band from very early on such that hearing was restricted to the frequency of the mating call/signal of its own species.

In a hypothetical scenario, acoustic communication in crickets may have evolved from a close range interaction of sender and receiver during which both mating partners may have been in close - probably antennal - contact. Under these circumstances the requirement for an ear was largely restricted to a given sensitivity to monitor the partners mating call, but the ability for localization was not necessary. With the advent of resonance the signalling range may have expanded dramatically such that females nearby, but without visual contact were able to detect a singing male easily. However, then females were faced with the task of localization that was as yet not - or only poorly - implemented. Therefore specific improvements such as the acoustic trachea that connects both ears and the septum in between were necessary in order to employ a pressure-difference receiver for localization [31]. Due to biophysical constraints a system for localization evolved that was tuned to a specific frequency [12,14,32,33].

Given this hypothetical evolutionary scenario, sensory structures as part of acoustic communication in crickets are highly dedicated sensory processing devices and not a sensory system that evolved to encode a large and divergent stimulus space (for hearing in grasshoppers see e.g. [34,35]; for hearing in vertebrates, see e.g. [36]). In crickets, given substructures responsible for sensitivity and localization may have evolved independently from one another and at different times, in order to fulfil their particular tasks.

(2) Local adaptation drives present configuration of sensory traits:

#### Allopatric setting

In allopatry, there is no constraint for females to discriminate towards other sibling species. If so, *G.b*. males may serve both sensory traits of females and produce rather intermediate frequencies. *G.c*. females revealed - possibly for reasons of low competition in central Europe by other cricket species - a large variability in their best frequencies which resulted in a wide rather than a narrowly tuned sensitivity filter in the mean (Fig. 1). Consequently, males direct their CF to serve the directionality trait of females, since there is little to optimize in terms of sensitivity. This bias is probably additionally driven by the extremely high IIDs that *G.c*. females reveal in their directionality trait.

#### Sympatric setting

Inspection of the geographic distribution of *T.o*. and *T.c*. suggests that *T.o*. moved from the origin of the genus Teleogryllus from southern Africa towards the East-Asia and is today a common cricket species in Indopacific regions with populations even in Hawaii [37,38]. When *T.o*. invaded Australia and moved towards southern, colder regions two life history traits may have evolved that eventually drove the speciation process towards *T.c*.: larger body size and a diapause [39]. In southern Queensland, both species occur sympatrically up to the present day [40]. Conceivably, selection on *T.c*. was stronger to discriminate towards its sibling species and thus directional selection on both sensory traits drove these to low frequency values (Figs. 5D, 6D) that were then obeyed by males. Consequently, male variation of the frequency trait is also much lower than in all other species investigated here, since both sensory traits almost coincide with their best frequency. A strong indication for directional selection by *T.c*. females is the unusually high roll-off of the high-frequency side of the sensitivity function (Fig. 2). Since the size differences between *T.c*. and *T.o*. are small, one may expect that both species possess rather similar optimal frequencies for efficient signalling [9]. Although *T.c*. is only slightly larger than *T.o*. (pronotum width 6.16 compared to 5.7 mm), and significantly smaller compared to *G.c*. (pronotum width 8.0 mm), males of *T.c*. nevertheless call at much lower frequencies of 4.0 kHz. This would indicate that *T.c*. deviated more from the frequency optimum expected from their body size. Interestingly, *T.o*. is the species with the strongest mismatch between the two filters, and by calling at the population mean CF of 4.8 kHz they sacrifice about 50% of the available directionality in female receivers. This might indicate that for species competing for neighbouring transmission channels, it is more adaptive to separate the two CFs and tune the filters accordingly in order to avoid signal interference. Only under circumstances of low or missing competition, it may be possible to shift the CF of the calling song to values intermediate between the two filters, as in *G.b*.

## Methods

### Animals

Four species of field cricket were used in our study. *G. bimaculatus *were obtained from a local supplier. As these animals were also subject of behavioural phonotactic experiments in a previous study [17], all twenty individuals were females. Fourteen G.c. were collected in the field in the vicinity of Graz; only one individual was a female. *T. oceanicus *(3 males, 8 females) and T.c. (7 males, 2 females) were obtained from our own culture at the Humboldt University. Although the majority of measurements was sex-biased (towards females in *G.b*. and males in *G.c*.) we are confident that sex differences in tuning and/or sensitivity do not exist, since data on *G.b*. from another study [41] in which both sexes were tested showed that neither the sensitivity nor the best frequency was sex-biased (t-test; p = 0.588 and 0.950, respectively). Also, the data on females available for the other 3 species show that their tuning and sensitivity, as well as directionality, are all within the range of variation of the males. *G.b., T.o*. and *T.c*. were raised to adulthood in the laboratory and were used for neurophysiology starting one week after the final moult, whereas *G.c*. was used not later than 3 days after they have been caught in the wild.

### Sound recording and analysis

Calling songs of a total of 40 individual males of *G.c*. were recorded in the field using a sound level meter (Rion NL-21) with an integrated microphone (UC-52) and a Sony professional walkman. Temperature during the field recordings varied from 17°C to 27°C. Similarly, calling songs of *G.b*. (N = 31), *T.o*. (N = 38), and *T.c*. (N = 12), were recorded in the laboratory from isolated males, at temperatures between 23 and 27°C. Songs were digitised using "Batsound" software at a sampling rate of 44 kHz and analysed with respect to the carrier frequency (CF) using cool edit software to the nearest 0.01 kHz.

### Neurophysiology

In order to determine the neuronal and directional tuning, neurophysiological experiments have been performed in an acoustically isolated Faraday-cage at room temperature between 21-23°C. Extracellular recordings of the discharges of a prominent auditory interneuron, the AN1-neuron, were used to examine both the frequency tuning of the afferent sensory system processing conspecific calling song, and for the peripheral directionality. Previous studies indicate that positive phonotaxis in field crickets is based on the activity of this pair of interneurons [42,43]. AN1 is more or less specifically tuned to the calling song frequency in all field cricket species examined, and the specific tuning in individual females strongly predicts positive phonotaxis in a two-choice paradigm [17]. Action potential activity was recorded with extracellular tungsten hook-electrodes placed at the connectives between prothoracic and suboesophageal ganglia. Insects were briefly anesthesized with chlorethylene and fixed ventral side up on a thin (1 mm) platform with dental wax. The forelegs were fixed in the natural walking position onto thin wires (diameter 0.6 mm). The preparation was sealed with petroleum jelly to prevent desiccation of the connectives. AN1-discharges were amplified using a custom-made amplifier, visualized on an oscilloscope (Agilent 54616B) and monitored through headphones. The threshold in response of a given stimulus was defined as the sound pressure level (SPL) which elicited at least one AP/syllable of calling song in at least three out of five stimulations.

### Controls for the opening of the acoustic spiracle

**Figure 7:**
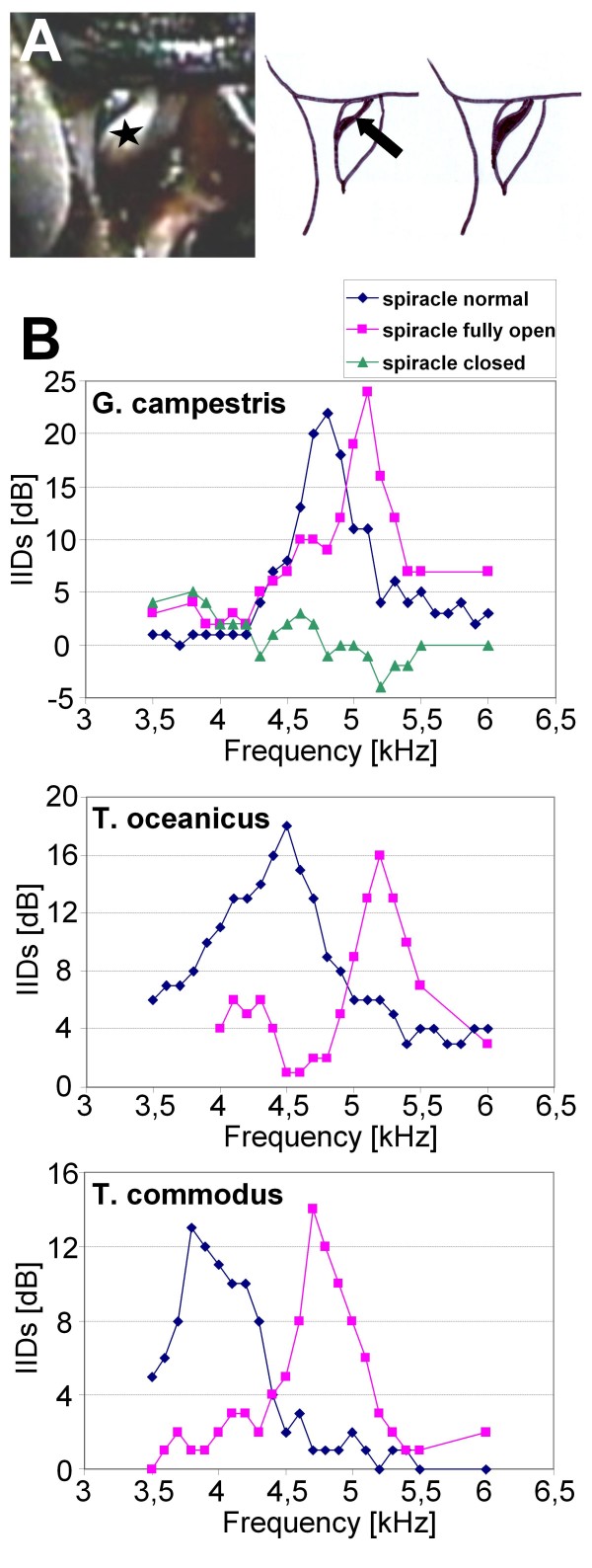
**The opening status of the acoustic spiracle and directional tuning**. **A**: Close-up of the right spiracle opening of a female *G.b*. while performing phonotaxis towards a model song. The cuticular flap (asterisk) leaves the acoustic spiracle only partially open. The opening status is passively modulated during the swing and stance phase of the foreleg, as indicated by the two sketches in A (the right one recorded during the swing phase; opening indicated by arrow). In all experiments, the directional tuning of the ear was monitored in an opening status of the spiracle as indicated in A. Only when the cuticular flap was completely removed this resulted in a shift of the best frequency in directional tuning towards unusual high frequencies in all 4 cricket species (here shown for *G.c*., *T.o*. and *T.c*.). Blocking of the spiracle with wax abolished the tuned directionality completely (only shown for *G.c*. in B).

Since the opening status of the acoustic spiracle may contribute to the observed variation in the frequency optimum of the peripheral directionality (see results) we monitored the spiracle in behaving animals on a trackball system described in detail by [17]. Close-up recordings were performed in three females using a micro-video camera (Toshiba IK-SM50H29) while the females performed phontaxis towards calling song models differing in CF from 4.0, 4.9 and 5.5 kHz. The opening of the spiracle is controlled by a cuticular flap (asterisk in Figure 7A), and this flap was only partially lifted in all three females under the different playback regimes. In no case we observed that the spiracle was fully open. Moreover, the opening status was modulated by the stepping cycle of the front legs to some degree (see Figure 7A). Therefore, in the neurophysiological experiments presented here, this status was controlled carefully and was kept similar and only partially open as observed in the behavioural trials. Only in control experiments with the cuticular flap completely removed, and thus the opening of the spiracle at the maximum, we observed a shift in the best frequency of directional hearing as compared to the control condition. In all experiments with the 4 species this resulted in a shift of the directional maximum towards unusually high frequencies (figure 7B). By contrast, closing the spiracle resulted in a complete loss of directionality (figure 7B) similar to what has been reported after destruction of the median septum [14].

### Playbacks

We used a standard temporal pattern as stimulus for all four species of field cricket, the CF of which was varied from 3.0 to 6 kHz, in increments of 0.5 kHz or 0.1 kHz. The rationale for using this narrow range of frequencies was that the natural variation in CF of the calling songs in the four species covers this range. Frequencies far outside this range, which never occur as CF in calling songs elicited "anomalous phonotaxis" in some females [44] were not included. We used the same temporal pattern of the stimulus despite the known differences in the songs between species because it was only used for threshold measurements, and thus possible variations in threshold due to different patterns were minimized. The standard stimulus had a pulse duration of 23 ms and pause duration of 16 ms, with four pulses per chirp. Four chirps with an interchirp pause of 230 ms were followed by a silent interval of 750 ms before this series of chirps was repeated in an endless loop. Sound stimuli were generated with Cool Edit software and broadcast by standard PC audio boards. A speaker (Raveland MHX 138) was placed at a distance of 40 cm, at an angular separation of 30° left or 30° right of the longitudinal body axis. These frontal angular positions of ± 30° are rather typical positions during the zig-zag phonotactic walk of a female and thus represent a useful measure of the directional gradient in the frontal position of the insect [12]. The model songs were amplified and attenuated in steps of 1dB using a Tucker-Davis system (Alachua, Florida). The tuning of AN1 was determined with ipsilateral stimulation at frequencies ranging from 3.5 to 6 kHz (in experiments with *T.c*. from 3.0 to 6.0 kHz), in increments of 100 or 500 Hz. In order to measure only the directionality provided by the anatomical arrangement of the acoustic tracheae in the periphery, inhibitory central nervous interactions were eliminated by cutting the contralateral leg nerve, which carries the fibers of the sensory cells in the ear. IIDs were calculated by measuring the thresholds of the AN1-neuron with ipsilateral and contralateral stimulation at an angular frontal deviation of ± 30°. The strongest directional gradient occurs within these angular positions [12], and more lateral stimulus angles only slightly increase IIDs, but do not change the frequency tuning of the pressure difference receiver. The threshold differences between ipsi- and contralateral stimulation represent the IID for a given frequency at a stimulation angle of ± 30°, similar to biophysical measurements using laser-vibrometry [14].

## Abbreviations

AN: ascending interneuron; CF: carrier frequency; *G.b*: Gryllus bimaculatus; *G.c*: Gryllus campestris; *T.c*: Teleogryllus commodus; *T.o*: Teleogryllus oceanicus; IID: interaural intensity difference; SPL: sound pressure level.

## Competing interests

The authors declare that they have no competing interests.

## Authors' contributions

KK, MH and HR conceived the study and equally participated in the interpretation of the results. MH provided specimen of *T.c*. and *T.o*. and the analysis of their calling songs. KK carried out the experiments and wrote the first draft of the manuscript. All authors read and approved the final manuscript.
